# Stress hyperglycemia ratio and the clinical outcome of patients with heart failure: a meta-analysis

**DOI:** 10.3389/fendo.2024.1404028

**Published:** 2024-07-05

**Authors:** Liang Li, Zhikun Zhao, Shasha Wang, Jiajia Wang

**Affiliations:** Department of Geriatric Medicine, Fourth Medical Center of PLA General Hospital, Beijing, China

**Keywords:** heart failure, stress hyperglycemia ratio, prognosis, mortality, meta-analysis

## Abstract

**Background:**

Stress hyperglycemia ratio (SHR) is a newly suggested measure of stress-induced hyperglycemia that combines both short-term and long-term glycemic conditions. The study aimed to explore the association between SHR and the incidence of adverse clinical events with heart failure (HF) through a meta-analysis.

**Methods:**

Cohort studies relevant to the aim of the meta-analysis were retrieved by search of electronic databases including PubMed, Web of Science, Embase, Wanfang, and CNKI. A random-effects model was used to combine the data by incorporating the influence of between-study heterogeneity.

**Results:**

Ten studies involving 15250 patients with HF were included. Pooled results showed that compared to patients with lower SHR at baseline, those with a higher SHR were associated with an increased risk of all-cause mortality during follow-up (risk ratio [RR]: 1.61, 95% confidence interval [CI]: 1.17 to 2.21, p = 0.003; I^2^ = 82%). Further meta-regression analysis suggests that different in the cutoff of SHR significantly modify the results (coefficient = 1.22, p = 0.02), and the subgroup analysis suggested a more remarkable association between SHR and all-cause mortality in studies with cutoff of SHR ≥ 1.05 than those with cutoff of SHR < 1.05 (RR: 2.29 versus 1.08, p for subgroup difference < 0.001). Subsequent meta-analyses also showed that a high SHR at baseline was related to the incidence of cardiovascular death (RR: 2.19, 95% CI: 1.55 to 3.09, p < 0.001; I^2^ = 0%), HF-rehospitalization (RR: 1.83, 95% CI: 1.44 to 2.33, p < 0.001; I^2^ = 0%), and major adverse cardiovascular events (RR: 1.54, 95% CI: 1.15 to 2.06, p = 0.004; I^2^ = 74%) during follow-up.

**Conclusion:**

A high SHR at baseline is associated with a poor clinical prognosis of patients with HF.

**Systematic review registration:**

https://inplasy.com, identifier INPLASY202430080.

## Introduction

Heart failure (HF) is a clinical condition characterized by the dysfunction of cardiac systolic and/or diastolic function, leading to inadequate perfusion for peripheral organs and tissues (1, 2). Due to worldwide population aging and advancements in treatments for different cardiovascular diseases, there is an anticipated growth in the number of HF patients globally (3, 4).

Hyperglycemia has been found to be influential in the pathogenesis and progression of HF (5, 6). In addition to chronic hyperglycemia, there is growing evidence indicating that stress-induced hyperglycemia (SIH), a physiological response to acute or severe illness like HF, could also be a crucial physiological mechanism contributing to the decline in cardiac function (7, 8). In individuals with HF, overly active neurohormonal factors such as catecholamine and inflammatory cytokines may trigger gluconeogenesis and glycogenolysis processes, leading to increased blood sugar levels (9, 10). Consequently, SIH further exacerbates myocardial damage and dysfunction through oxidative stress, inflammatory reaction, and vascular endothelial dysfunction (11–13).

Previous clinical studies examining the link between SIH indicated by admission blood glucose (ABG) and the prognosis of HF patients have yielded conflicting findings (14–17). One predominant reason for this is that an increase in ABG may not accurately reflect SIH in many patients, as it does not account for chronic glycemic status (18). Recent research proposes that the stress hyperglycemia ratio (SHR), calculated as the ratio of ABG to the average chronic glucose level estimated by hemoglobin A1c (HbA1c), could offer a more precise definition of SIH by considering both acute and chronic glycemic status (19, 20). A recent meta-analysis found that higher SHR values were linked to a significantly increased risk of mortality in patients with myocardial infarction (21). However, due to inconsistent results from previous studies (22–31), it remains uncertain whether SHR relates to the prognosis of HF patients. Accordingly, in this study, a meta-analysis to was conducted to systematically assess the impact of SHR on clinical outcomes, such as all-cause mortality among HF patients, and to determine the potential influences of the cutoff of SHR on the association between SHR and all-cause mortality of patients with HF.

## Materials and methods

The 2020 Preferred Reporting Items for Systematic reviews and Meta-Analyses statement (32, 33) was followed in this study. The Cochrane Handbook (34) for systematic review and meta-analysis was referenced throughout the study. The meta-analysis protocol was registered on the International Platform of Registered Systematic Review and Meta-Analysis Protocols with the registration number INPLASY202430080.

### Literature analysis

Three main electronic databases including PubMed, Web of Science, Embase, Wanfang, and China National Knowledge Infrastructure (CNKI) were used for literature search with a predefined combined search term including (1) “stress hyperglycemia” OR “stress hyperglycaemia” OR “stress-induced hyperglycemia” OR “stress induced hyperglycemia” OR “stress-induced hyperglycaemia” OR “stress induced hyperglycaemia” OR “admission hyperglycemia” OR “admission hyperglycaemia” OR “admission glucose” OR “stress hyperglycemia ratio” OR “stress hyperglycaemia ratio” OR “glycemic ratio” OR “stress-hyperglycaemia ratio” OR “stress-hyperglycemia ratio” OR “glycemic gap” OR “relative hyperglycemia” OR “acute-to-chronic glycemic ratio”; (2) “heart failure” OR “cardiac failure” OR “cardiac dysfunction”; and (3) “mortality” OR “death” OR “hospitalization” OR “rehospitalization” OR “prognosis” OR “survival” OR “major adverse cardiovascular events” OR “MACE”. Only studies with human subjects and published in English or Chinese were included. A manual screening of the citations from the pertinent articles and reviews (cross-references) was also carried out for possible eligible studies. The conclusive database search took place on February 12, 2024.

### Inclusion and exclusion criteria

The inclusion criteria were made according to the PICOS principle

1. P (patients): Patients with confirmed diagnosis of HF.
2. I (exposure): The SHR was calculated as the ratio of admission glucose to the average chronic glucose level (admission glucose [mmol/L]/(1.59 x HbA1c[%] - 2.59). The cutoff for the defining a high SHR was consistent with the value which was used in the original studies.
3. C (control): Patients with a low level of SHR at baseline was considered as the controls.
4. O (outcome): The primary outcome of the meta-analysis was the incidence of all-cause mortality during follow-up compared between HF patients with higher versus lower category of SHR at baseline. The secondary outcomes were the incidence of cardiovascular (CV) death, HF-rehospitalization, and the composite outcome of major adverse cardiovascular events (MACE) during follow-up. No restriction for the length of follow-up duration was applied in this study.
5. S (study design): Cohort studies, including the prospective and retrospective cohort studies, published as full-length articles in peer-reviewed journals.

We excluded reviews, meta-analyses, studies with SHR analyzed as continuous only, or studies without outcomes of interest. In cases where there was potential overlap in patient population across multiple studies, only the study with the largest sample size was included in this analysis.

### Data collection and quality assessment

Two separate authors conducted a thorough search of academic literature, performed data collection and analysis, and independently assessed the quality of the studies. Any discrepancies that arose were resolved by involving the corresponding author in discussion for final decision-making. Data on study information, design, diagnosis of the patients, sample size, age, sex, and diabetic status of the patients, the cutoffs of SHR, follow-up durations, outcomes evaluated, and variables adjusted in the regression model for studying the association between SHR and clinical outcomes of patients with HF were gathered. The assessment of study quality was carried out using the Newcastle-Ottawa Scale (NOS) (35), which involved scoring based on criteria including participant selection process, comparability among groups, and validity of outcomes. This scale utilized a rating system ranging from 1 to 9 stars; higher stars indicated better study quality.

### Statistical methods

An association between SHR and the clinical outcomes of patients with HF was presented using risk ratio (RR) and corresponding 95% confidence interval (CI), compared between HF patients with high versus low SHR at baseline. For studies reporting odds ratio (OR), data were converted to RRs for the meta-analysis (RR=OR/([1−pRef]+[pRef×OR]), where pRef is the prevalence of the outcome in the reference group (a low SHR group) (36). Data of RRs and standard errors were calculated based on the 95% CIs or p values, followed by a logarithmical transformation to ensure stabilized variance and normalized distribution (34). We combined the log RR or log hazard ratios (HR) and corresponding standard errors by the inverse variance approach. The heterogeneity among studies was assessed using the Cochrane Q test and I^2^ statistic (37, 38), with I^2^ > 50% indicating significant statistical heterogeneity. A random-effects model was used for result aggregation considering the influence of heterogeneity (34). For the primary outcome of all-cause mortality, the sensitivity analysis by omitting one study at a time was performed to evaluate the robustness of the finding. For characteristics presented as the continuous variables, such as sample size, mean age, proportion of men, proportion of diabetic patients, cutoff of SHR, follow-up duration, and study quality scores, a univariate meta-regression analysis was also performed (34) to explore the influence of these variables on the outcome of the meta-analysis. For the continuous variables, medians were directly retrieved as means in the meta-regression analysis. Additionally, multiple subgroup analyses were performed to evaluate the influences of study characteristics on the results, such as in acute or chronic HF, in diabetic or non-diabetic patients, as well as subgroup analyses according to the cutoffs of SHR and follow-up durations of the included studies. Medians of continuous variables were selected as the cutoff values for defining subgroups. Publication bias estimation involved constructing funnel plots initially evaluated through visual inspection for symmetricity before being analyzed using Egger’s regression test (39), where a p < 0.05 indicates statistical significance. These analyses were conducted using the RevMan Version 5.1 (Cochrane Collaboration, Oxford, UK) and Stata software version 17 (Stata Corporation, College Station, TX).

## Results

### Study inclusion

**Figure 1:**
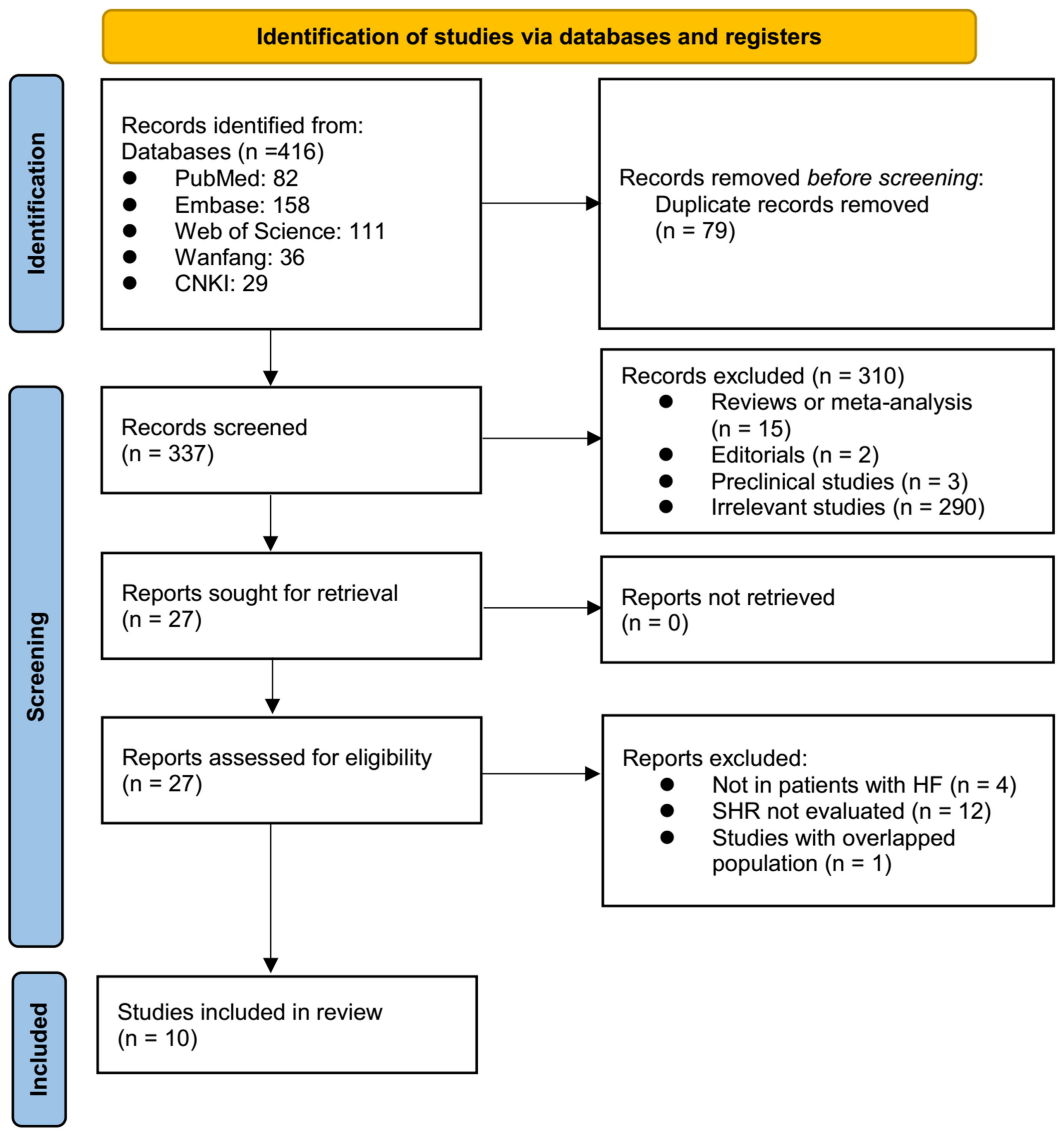
Process of conducting literature search and identifying studies.

The process of selecting relevant studies for inclusion in the meta-analysis is depicted in **Figure 1**. Initially, 416 potentially pertinent records were identified through thorough searches of five databases. Among these, 79 were removed due to duplication. Subsequent screening based on the titles and abstracts resulted in the exclusion of an additional 310 studies that did not align with the aim of the meta-analysis. The full texts of the remaining 27 records underwent independent review by two authors, leading to the removal of a further 17 studies for various reasons detailed in **Figure 1**. Ultimately, ten cohort studies remained (22–31) and were considered suitable for subsequent quantitative analyses.

### Overview of the studies’ characteristics

**Table 1** presents the summarized characteristics of the included studies. Overall, one prospective cohort (23) and nine retrospective cohort studies (22, 24–31) were included in the meta-analysis. These studies were published between 2021 and 2024, and performed in China, Spain, Portugal, and the United States. All of the studies included adult populations with HF. The mean ages of the patients were 58.2 to 83.0 years. Methods for defining the cutoff of SHR varied among the included studies, such as the use of the median (22), tertiles (23, 25, 26), quintiles of SHR (28), receiver operating characteristic analysis derived cutoffs (24, 27, 30, 31), or arbitrarily determined cutoff (29). The cutoff values for defining a high SHR varied from 0.82 to 1.75 among the included studies. The follow-up durations varied from within hospitalization to 48 months. The primary outcome of all-cause mortality was reported in ten cohorts (1984 events) (22–31), while the secondary outcomes of CV death (28, 30) were reported in two studies (227 events) (28, 30), HF rehospitalization in two studies (346 events) (28, 30), and MACE in three studies (360 events) (24, 25, 27), respectively. Multivariate analyses were used in all of the included studies when the association between SHR and the clinical outcomes of patients with HF was reported, and variables such as age, sex, hemodynamic parameters, comorbidities, ejection fraction (EF), and concurrent medications were adjusted to a varying extent among the included studies. The data of OR was reported in five studies (22, 24, 25, 27, 29), and the data of HR was reported in the other five studies (23, 26, 28, 30, 31). The NOS of the included studies were seven to nine stars, suggesting overall good study quality (**Table 2**).

**Table 1:** Characteristics of the included studies.

Study	Location	Design	Diagnosis	Sample size	Mean age (years)	Male (%)	DM (%)	Methods for defining SHR category	Cutoff for defining a high SHR	Follow-up duration (months)	Outcomes reported (Number of events)	Variables adjusted
Wu 2021 (22)	China	RC	AHF	307	83	49.8	39.7	Median	1.04	12	All-cause mortality (89)	Age, sex, BMI, HR, SBP, LVEF, NYHA class, HGB, SCr, NT-proBNP, DM, HTN, CAD, COPD, and concurrent medications
Zhou 2022 (25)	China	RC	HF	2875	71.2	61.3	100	Tertiles	1.09	Within hospitalization	All-cause mortality (80); MACE (154)	Age, sex, SBP, eGFR, NT-proBNP, admission department, CCI, ischemic etiology, insulin use, and venous loop diuretics at baseline
Carrera 2022 (23)	Spain	PC	AHF	1062	72.6	56.7	55.6	Tertiles	0.82	48	All-cause mortality (546)	Age, sex, BMI, smoking, HTN, dyslipidemia, CKD, HGB, NT-proBNP, LVEF, NYHA class, ischemic etiology, PAD, and COPD
Xiao 2022 (24)	China	RC	AHF	106	68.5	63.2	37.7	ROC analysis derived	1.05	Within hospitalization	All-cause mortality (21); MACE (48)	Age, sex, LVEF, NT-proBNP, cardiac shock, and WBC
Zhou 2023 (28)	China	RC	AHF	780	68.9	63.3	100	Quintile	1.14	39	All-cause mortality (169); CV death (165); HF rehospitalization (231)	Age, sex, smoking, BMI, NT-proBNP, TG, LDL-C, SCr, SBP, LVEF, CAD, AF, and concurrent medications
Cunha 2023 (26)	Portugal	RC	AHF	599	72	55.9	50.9	Tertiles	0.88	3	All-cause mortality (102)	Age, sex, LVEF, ischemic etiology, eGFR, and concurrent medications
Zhai 2023 (27)	China	RC	AHF	755	75.1	57.4	36.2	ROC analysis derived	1.23	1	All-cause mortality (79); MACE (158)	Age, sex, BMI, HTN, DM, CAD, LVEF, BNP, and cardiac shock
Mohammed 2024 (30)	China	RC	HFpEF	400	71	56.5	41.5	ROC analysis derived	0.99	41	All-cause mortality (75); CV death (62); HF rehospitalization (115)	Age, sex, BMI, smoking, NYHA class, AF, HTN, CKD, DM, TC, LDL-C, eGFR, CRP, NT-proBNP, LVEF, and concurrent medications
Li 2024 (29)	USA	RC	CHF	8268	72.4	56.4	56.7	Arbitrary determined	1.75	Within hospitalization	All-cause mortality (792)	Age, sex, HTN, DM, AF, acute HF, MI, stroke, ischemic cardiomyopathy, CKD, NT-proBNP, SCr, BUN, and concurrent treatments
Shao 2024 (31)	China	RC	AHF	98	58.2	38.8	19.4	ROC analysis derived	1.17	3	All-cause mortality (31)	Age, sex, BMI, HR, SBP, LVEF, HTN, LDL-C, serum sodium, SCr, CRP, and LVEF

DM, diabetes mellitus; SHR, stress hyperglycemia ratio; RC, retrospective cohort; PC, prospective cohort; CHF, chronic heart failure; AHF, acute heart failure; HFpEF, heart failure with preserved ejection fraction; ROC, receiver operating characteristic; CV, cardiovascular; MACE, major adverse cardiovascular events; BMI, body mass index; SBP, systolic blood pressure; DBP, diastolic blood pressure; HR, heart rate; CRP, C-reactive protein; eGFR, estimated glomerular filtrating rate; NT-proBNP, N-terminal pro B-type natriuretic peptide; HbA1c, hemoglobin A1c; LVEF, left ventricular ejection fraction; AF, atrial fibrillation; NYHA, New York Heart Association; BNP, B-type natriuretic peptide; CAD, coronary artery disease; HGB, hemoglobin; COPD, chronic obstructive pulmonary disease; HTN, hypertension; SCr, serum creatinine; CCI, Charlson Comorbidity Index; PAD, peripheral artery disease; WBC, white blood cell; TG, triglyceride; LDL-C, low-density lipoprotein cholesterol; TC, total cholesterol; MI, myocardial infarction; CKD, chronic kidney disease.

**Table 2:** Study quality evaluation via the Newcastle-Ottawa scale.

Study	Representativeness of the exposed cohort	Selection of the non-exposed cohort	Ascertainment of exposure	Outcome not present at baseline	Control for age and sex	Control for other confounding factors	Assessment of outcome	Enough long follow-up duration	Adequacy of follow-up of cohorts	Total
Wu 2021 (22)	0	1	1	1	1	1	1	1	1	8
Zhou 2022 (25)	0	1	1	1	1	1	1	0	1	7
Carrera 2022 (23)	1	1	1	1	1	1	1	1	1	9
Xiao 2022 (24)	0	1	1	1	1	1	1	0	1	7
Zhou 2023 (28)	1	1	1	1	1	1	1	1	1	9
Cunha 2023 (26)	0	1	1	1	1	1	1	0	1	7
Zhai 2023 (27)	0	1	1	1	1	1	1	0	1	7
Mohammed 2024 (30)	0	1	1	1	1	1	1	1	1	8
Li 2024 (29)	0	1	1	1	1	1	1	0	1	7
Shao 2024 (31)	0	1	1	1	1	1	1	0	1	7

### Meta-analysis for the association between SHR and all-cause mortality

**Figure 2:**
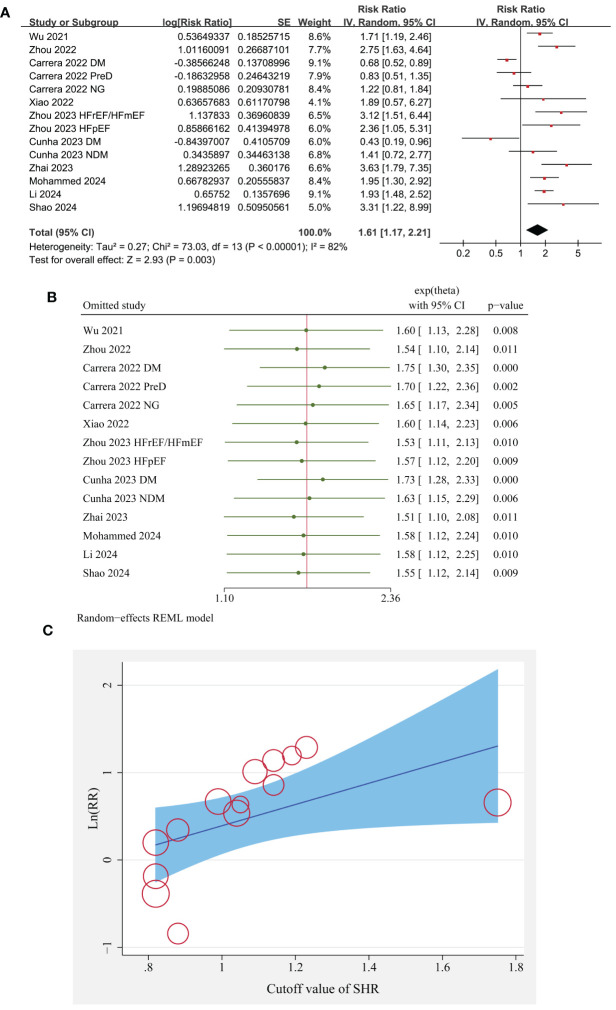
The overall meta-analysis, sensitivity, and meta-regression analyses of the association between SHR and all-cause mortality of patients with HF; **(A)** forest plots for the overall meta-analysis of the association between SHR and all-cause mortality; **(B)** results of sensitivity analyses association between SHR and all-cause mortality; and **(C)** the univariate met-regression analysis for the influence of SHR cutoff on the association between SHR and all-cause mortality of patients with HF.

Since two studies reported the outcome according to the diabetic status of the patients (23, 26), and another study reported the outcome according to the EF of the patients (28), these datasets were included in the meta-analysis independently. Overall, pooled results of 14 datasets from ten cohort studies showed that compared to patients with lower SHR at baseline, those with a higher SHR were associated with an increased risk of all-cause mortality during follow-up (RR: 1.61, 95% CI: 1.17 to 2.21, p = 0.003; **Figure 2A**) with significant heterogeneity (I^2^ = 82%).

Sensitivity analysis by excluding one dataset at a time did not significantly change the results (p all < 0.05; **Figure 2B**).

Further meta-regression analysis suggests that different in the cutoff of SHR significantly modified the association between SHR and all-cause mortality of patients with HF (coefficient = 1.22, p = 0.02; **Table 3**; **Figure 2C**), but not for the other variables such as sample size, mean age, proportion of men, percentile of diabetic patients, follow-up duration, or study quality scores (p all > 0.05; **Table 3**).

**Table 3:** Univariate meta-regression analysis for the outcome of all-cause mortality.

Variables	RR for the association between SHR and all-cause mortality of HF patients		
	Coefficient	95% CI	P values
Sample size	0.000029	-0.000139 to 0.000196	0.72
Mean age (years)	-0.028	-0.105 to 0.048	0.43
Men (%)	0.0027	-0.0661 to 0.0715	0.93
Diabetes (%)	0.0045	-0.0113 to 0.0202	0.55
Cutoff of SHR	1.22	0.13 to 2.30	0.02
Follow-up duration (months)	-0.0097	-0.0243 to 0.0049	0.19
NOS	-0.19	-0.58 to 0.20	0.31

SHR, stress hyperglycemia ratio; RR, risk ratio; CI, confidence interval; HF, heart failure; NOS, Newcastle-Ottawa Scale.

**Figure 3:**
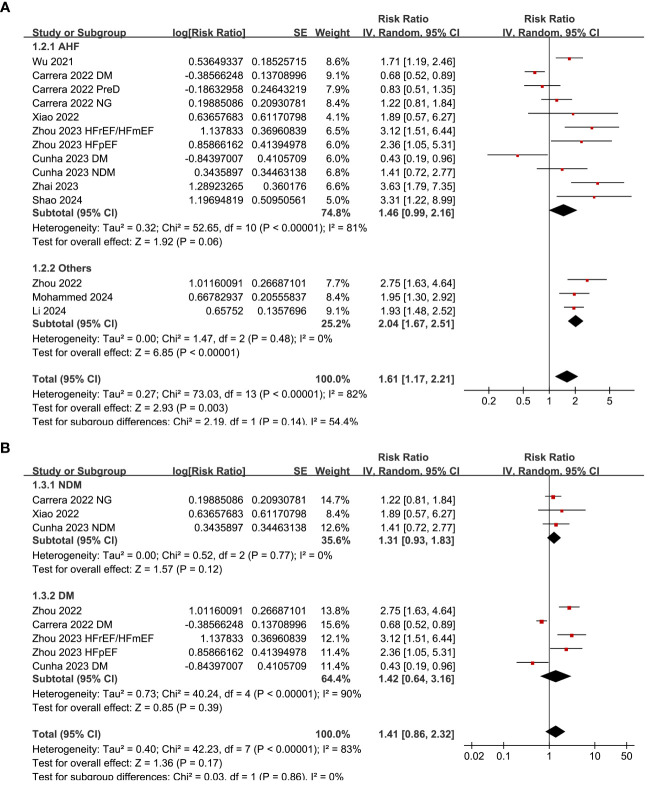
Forest plots for the subgroup analyses of the association between SHR and all-cause mortality of patients with HF; **(A)** forest plots for the subgroup analysis in acute HF and other HF; and **(B)** forest plots for the subgroup analysis in patients with and without diabetes.

**Figure 4:**
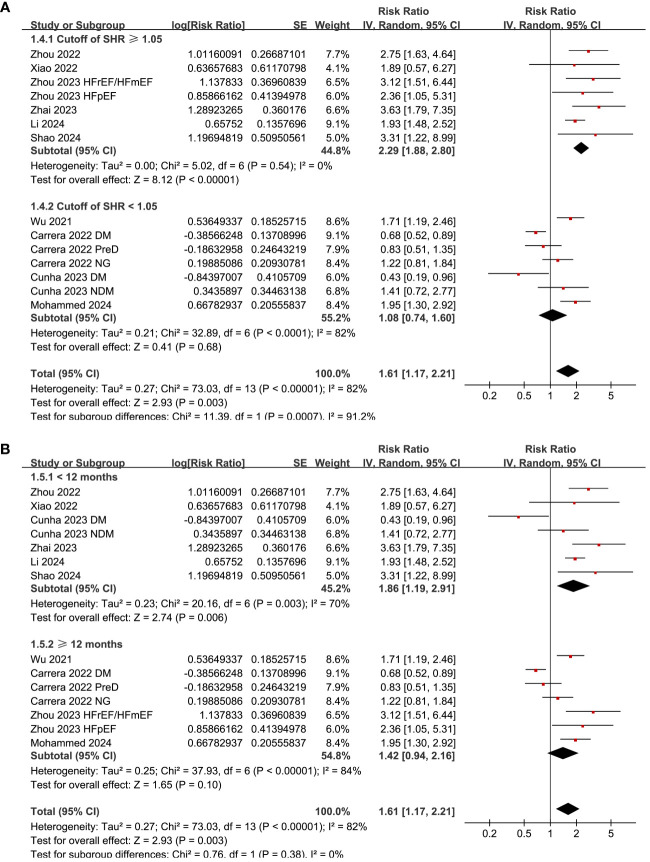
Forest plots for the subgroup analyses of the association between SHR and all-cause mortality of patients with HF; **(A)** forest plots for the subgroup analysis according to the cutoff of SHR; and **(B)** forest plots for the subgroup analysis according to the study quality scores.

Subgroup analysis suggested consistent association between a high SHR and an increased risk mortality in patients with acute HF and chronic/overall HF patients (p for subgroup difference = 0.14; **Figure 3A**), and in patients with and without diabetes (p for subgroup difference = 0.86; **Figure 3B**). Consistent to the results of meta-regression analysis, the subgroup analysis suggested a more remarkable association between SHR and all-cause mortality in studies with cutoff of SHR ≥ 1.05 than those with cutoff of SHR < 1.05 (RR: 2.29 versus 1.08, p for subgroup difference < 0.001; **Figure 4A**). Subgroup analysis showed similar results in studies with follow-up duration < 12 months and ≥ 12 months (p for subgroup difference = 0.38; **Figure 4B**).

### Meta-analysis for the association between SHR and other clinical outcomes

**Figure 5:**
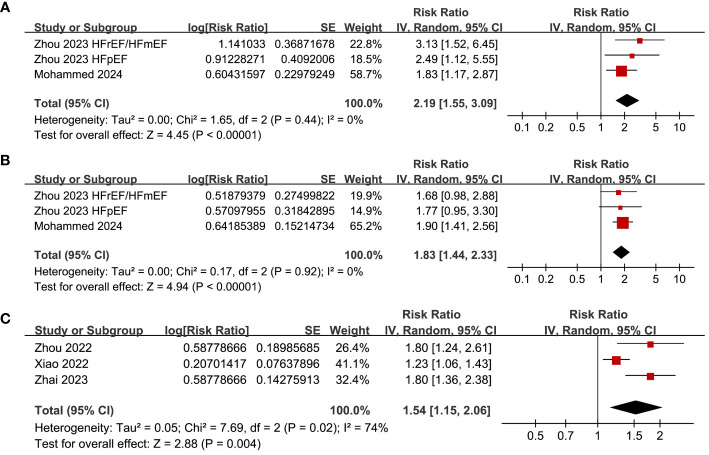
Forest plots for the meta-analysis of the association between SHR and the other clinical outcomes of patients with HF; **(A)** forest plots for the meta-analysis of the association between SHR and CV death; **(B)** forest plots for the meta-analysis of the association between SHR and HF-rehospitalization; and **(C)** forest plots for the meta-analysis of the association between SHR and MACE.

Pooled results of three datasets from two (28, 30), two (28, 30) and three studies (24, 25, 27) respectively showed that a high SHR at baseline was related to the incidence of CV death (RR: 2.19, 95% CI: 1.55 to 3.09, p < 0.001; I^2^ = 0%; **Figure 5A**), HF-rehospitalization (RR: 1.83, 95% CI: 1.44 to 2.33, p < 0.001; I^2^ = 0%; **Figure 5B**), and MACE (RR: 1.54, 95% CI: 1.15 to 2.06, p = 0.004; I^2^ = 74%; **Figure 5C**) of patients with HF during follow-up.

### Publication bias

**Figure 6:**
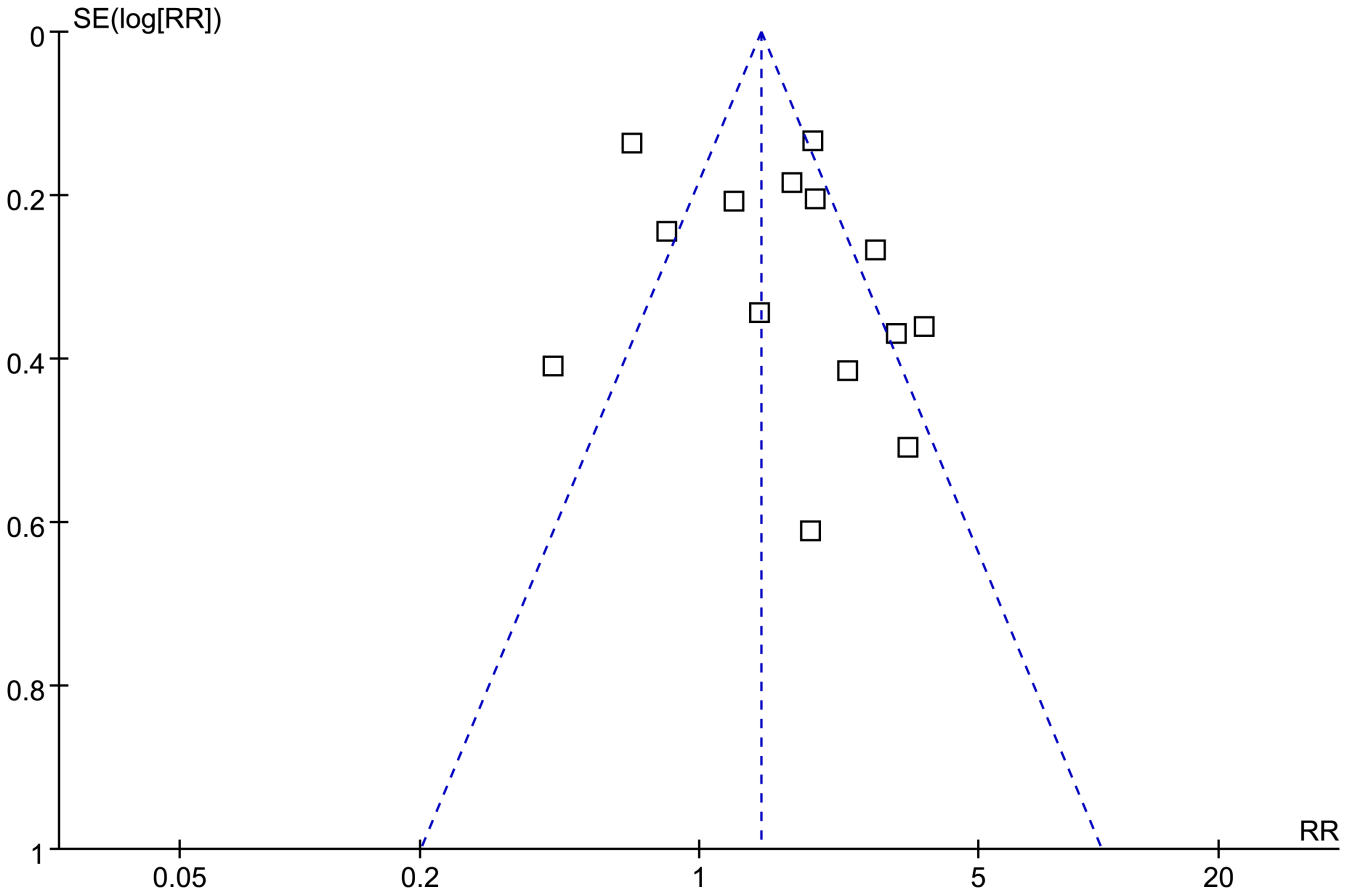
Funnel plots for the publication bias underlying the meta-analysis of the association between SHR and all-cause mortality of patients with HF.

The funnel plots for the meta-analysis of the association between SHR and all-cause mortality of HF are shown in **Figure 6**. The symmetrical nature of the funnel plots suggested the low likelihood of publication bias. The Result of the Egger’s regression test also showed a low risk of publication bias (p = 0.98). The publication biases underling the meta-analyses for the three secondary outcomes could not be determined because only three datasets were included.

## Discussion

This meta-analysis involved ten cohort studies and indicated that patients with HF and an elevated SHR upon admission faced a higher risk of all-cause mortality over the follow-up period. The sensitivity analysis, which involved excluding one dataset at a time, consistently produced similar results. Additionally, further examination through meta-regression and subgroup analysis suggested that the cutoff value for SHR could potentially influence the association between SHR and all-cause mortality in patients with HF, thus contributing to variations across studies. Notably, it was proposed that studies using a cutoff of SHR ≥ 1.05 showed a more significant association with all-cause mortality compared to those using a cutoff of SHR < 1.05. Additionally, the connection between SHR and patient mortality in HF appeared to remain unaffected by other study factors such as sample size, patient age, type of HF, diabetic status, follow-up duration, and study quality. Further investigation indicated that a high baseline SHR in HF patients was associated with higher risk of CV death, rehospitalization for HF, and MACE during follow-up. In summary, this meta-analysis implies a potential link between elevated SHR and adverse clinical results in HF patients.

This meta-analysis is possibly the first to thoroughly assess the correlation between SHR upon admission and the clinical results of HF patients. It’s crucial to recognize the strengths in methodology prior to interpreting the findings. We conducted a comprehensive search across five widely used English and Chinese electronic databases, identifying ten pertinent cohort studies for this analysis. By exclusively incorporating cohort studies, the meta-analysis was able to establish a longitudinal link between high SHR and adverse prognosis for these patients. Additionally, all of the included studies performed multivariate analyses when estimating the association between SHR and all-cause mortality of HF patients. These findings suggest a potentially independent relationship between elevated SHR and an increased likelihood of all-cause mortality in HF patients. Additionally, further subgroup and meta-regression analyses offered additional evidence supporting the strength of the association between high SHR and heightened risk of overall mortality in HF patients. Lastly, despite incorporating a limited number of studies, our meta-analyses also suggested that a high SHR in HF patients at baseline was associated with a higher probability of CV death, rehospitalization due to HF, and occurrence of MACE over the follow-up period. In conclusion, these results imply that a high SHR may function as an indicator of unfavorable prognosis for individuals with HF. These findings expanded the previous knowledge that SHR may be a risk factor of poor prognosis of patients with myocardial infarction (21), and even in patients with myocardial ischemia and nonobstructive coronary arteries (40).

The link between a high SHR and unfavorable prognosis for HF patients may indicate the significant role of SIH in the progression of HF. With both acute and chronic glycemic status taken into account, SHR is proposed to be more accurate than ABG in reflecting the severity of SIH (7). Pathophysiologically, SIH may further cause oxidative stress, inflammation, endothelial dysfunction, microvascular injury, and pro-thrombotic status, which may subsequently acerbate the impaired myocardial function (41). Conversely, deteriorated cardiac function enhances the activation of neurohormonal factors and inflammatory response, resulting in an increased blood glycemic level via gluconeogenesis and glycogenolysis, which forms a so-called vicious cycle (42). These could be the reasons why a high SHR is linked to poor prognosis for HF patients. The outcomes of the meta-regression analysis indicated that the threshold value for SHR in the studies included could impact the connection between SHR and all-cause mortality. The subgroup analysis revealed a more pronounced relationship between SHR and all-cause mortality in studies with an SHR cutoff ≥ 1.05 compared to those with a cutoff of < 1.05, suggesting a potential dose-dependent association with important implications for clinical practice. Incorporating SHR into the risk assessment of HF patients is important due to its invasive, convenient, and cost-effective nature. It is also crucial to investigate whether reducing SHR in HF patients can enhance prognosis, particularly for those with AHF. If this is the case, SHR could potentially be a more effective treatment target than simple plasma glucose. Future studies are needed to address these queries.

The research has certain limitations. Nine of the analyzed studies were carried out retrospectively, which could have introduced biases in selection and recall that might have impacted the outcomes. In addition, a high statistical heterogeneity (I^2^ = 82%) was observed among the included studies. Although our meta-regression and subgroup analysis suggested that difference in the cutoff SHR may be an important source of heterogeneity, other variations in studies characteristics, such as the comorbidities of the patients, concurrent treatments and medications, and the different follow-up durations may also contribute to the heterogeneity. Moreover, while multivariate analyses were conducted in all the included studies, it is still possible that unadjusted confounding factors may have influenced the association. Lastly, our reliance solely on observational research means that a conclusive causal relationship between high SHR and negative prognosis for patients with heart failure could not be definitively confirmed.

## Conclusions

The results of the meta-analysis suggest that HF patients with a high SHR at baseline may have an increased risk of adverse clinical outcomes over time, compared to those with a low SHR. More validation through comprehensive prospective studies and investigation into the underlying mechanisms is necessary. Considering its convenience and cost-effectiveness, these findings endorse the potential use of SHR as a prognostic indicator for HF patients.

## Data availability statement

The original contributions presented in the study are included in the article/supplementary material. Further inquiries can be directed to the corresponding authors.

## Author contributions

LL: Conceptualization, Data curation, Formal analysis, Investigation, Methodology, Validation, Visualization, Writing – original draft, Writing – review & editing. ZZ: Data curation, Formal analysis, Investigation, Validation, Visualization, Writing – original draft, Writing – review & editing. SW: Conceptualization, Data curation, Formal analysis, Investigation, Methodology, Resources, Software, Supervision, Validation, Writing – review & editing. JW: Data curation, Formal analysis, Investigation, Methodology, Validation, Visualization, Writing – review & editing.
